# CBMC: A Biomimetic Approach for Control of a 7-Degree of Freedom Robotic Arm

**DOI:** 10.3390/biomimetics8050389

**Published:** 2023-08-25

**Authors:** Qingkai Li, Yanbo Pang, Yushi Wang, Xinyu Han, Qing Li, Mingguo Zhao

**Affiliations:** 1 Department of Automation, Tsinghua University, Beijing 100084, China; 2 Beijing Innovation Center for Future Chips, Tsinghua University, Beijing 100084, China

**Keywords:** brain-inspired computing system, neuromorphic computing, spiking neural network, reinforcement learning, robotic arm

## Abstract

Many approaches inspired by brain science have been proposed for robotic control, specifically targeting situations where knowledge of the dynamic model is unavailable. This is crucial because dynamic model inaccuracies and variations can occur during the robot’s operation. In this paper, inspired by the central nervous system (CNS), we present a CNS-based Biomimetic Motor Control (CBMC) approach consisting of four modules. The first module consists of a cerebellum-like spiking neural network that employs spiking timing-dependent plasticity to learn the dynamics mechanisms and adjust the synapses connecting the spiking neurons. The second module constructed using an artificial neural network, mimicking the regulation ability of the cerebral cortex to the cerebellum in the CNS, learns by reinforcement learning to supervise the cerebellum module with instructive input. The third and last modules are the cerebral sensory module and the spinal cord module, which deal with sensory input and provide modulation to torque commands, respectively. To validate our method, CBMC was applied to the trajectory tracking control of a 7-DoF robotic arm in simulation. Finally, experiments are conducted on the robotic arm using various payloads, and the results of these experiments clearly demonstrate the effectiveness of the proposed methodology.

## 1. Introduction

The past few years have seen a blossoming of robotic applications in various fields, including manufacturing, health care and customer service, etc. The key issue of developing robots up to these applications lies in the control ability of the manipulation. For a force-controlled robot, the mapping between joint torque commands and the end-effector position is often generated by a previously acquired dynamic model, whose accuracy plays a vital role in the control ability. However, due to the uncertainties of the working environment and the development of elastic, muscle-like actuators, the accurate modeling of a robot’s dynamics is almost intractable in many scenarios. As a result of the evolution after billions of years, animals, especially human beings, have developed an adaptive control solution for motor performance that will work robustly in different environments with elastic muscles and joints, without the presence of a dynamic model, and can almost outperform the most state-of-art robots in many aspects. Therefore, researchers turn to bio-inspired approaches for inspiration.

Mimicking the learning ability of the cerebral cortex, some researchers have adopted artificial neural networks (ANNs), which are built by layers of computing neurons, as a solution for controlling robots without a dynamic model. In [1], an ANN-based control strategy is proposed for a flexible robotic arm with consideration of friction for both motor and payload. In the research of [2], an adaptive control method is introduced to the manipulator with unknown system dynamics. Wang et al. [3] take the output nonlinearity and unmodeled dynamics into consideration and develops an ANN module to approximate the unknown dynamics. In [4,5,6], some controlling methods based on ANN are also introduced to handle environment uncertainties and disturbance, like robotic manipulators working underwater. However, ANNs can only loosely model the functioning of the cerebral cortex. Artificial neurons in the network process information by nonlinear function of the sum of neuron inputs, and the output is transmitted through neuron connections and adjusted as the learning proceeds. These conventional neurons lack the ability to carry time-related information and thus the network can hardly deal with the temporal–spatial information of a robot during movement. Furthermore, the training process of ANNs, which is time and energy costing and computationally expensive, raises the stringency of demand on robotic processors [7].

To make up for the deficit, many other researchers have turn to spiking neural networks (SNNs) that mimic the underlying mechanisms of the brain more realistically. Unlike conventional neurons in ANNs, spiking neurons in SNNs precisely model the information transfer and processing as it happens in biological neurons, i.e., via discrete spikes that fire in certain timing patterns. This temporal coding mechanism in SNNs enables them to capture the temporal evolution of analog signals, making it a better solution for robotic control. Many works on SNN-based robotic applications have been presented. In [8,9], an SNN is trained with reinforcement learning to control a single-joint arm for target reaching. In [10,11], a 4-DoF robotic arm is controlled by a single-layer SNN network that is trained with spiking timing-dependent plasticity (STDP). Recently, DeWolf et al. [12] combined SNN and a neuromorphic chip to present a neurorobotic controller.

Despite the cerebral cortex, the involvement of the cerebellum in muscle and motor control has also been long advocated. Following this path, an SNN with cerebellum-inspired structure is presented in [13] for controlling a 2-DoF robotic arm, based on which a solution for compliant control and control under nondeterministic time delay is presented in [14,15], respectively.

The above studies have taken a positive step toward bio-inspired control in robots, mimicking some parts of the human brain in function or structure. However, their problem lies in viewing the cerebral cortex or the cerebellum as a stand-alone controller. An important observation about the brain is that schemas are distributed and computed in different brain areas. Motor control in vertebrates by the central nervous system (CNS) involves the cerebral motor cortex, basal ganglia, thalamus, cerebellum, brain stem, and spinal cord, and they work in collaboration in a hierarchical control loop [16]. It is therefore of great practical importance to study how this human control loop as a whole can be applied to robotic control.

In this paper, the main contributions are as follows:

- We propose a system model of the CNS-based Biomimetic Motor Control (CBMC) inspired by the human control loop for issues in control.
- A proposed implementation of this model involves utilizing an SNN for the cerebellum module, which is supervised by an ANN in the cerebral motor cortex module. This implementation is then applied to the control of a 7-DoF robotic arm.

The remainder of this paper is organized as follows: Section 2 presents the system model of CBMC. Section 3 will apply the above system to a 7-DoF robotic arm for demonstration. The results and discussion will be given in Section 4, and concluding remarks will be presented in Section 5.

## 2. CBMC: A Biomimetic Control Approach

**Figure 1:**
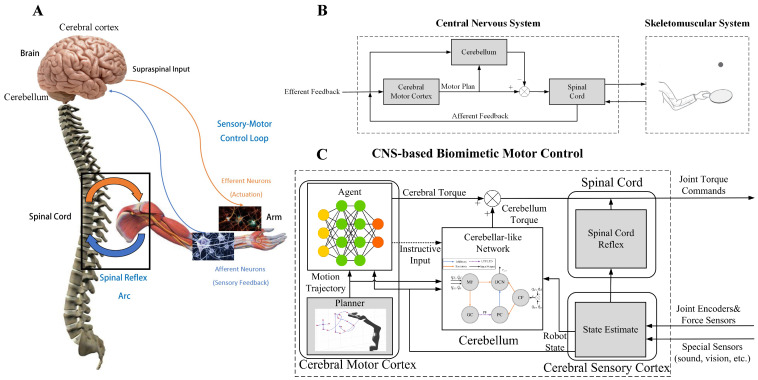
(**A**) Natural human motor control system. (**B**) Simplified human control loop related to the cerebral cortex, cerebellum, and spinal cord. (**C**) The CNS-based Biomimetic Motor Control (CBMC) control loop.

In human motor control, several areas of the CNS, including the cerebral cortex, cerebellum, and spinal cord, contribute to the temporal–spatial coordination of the skeletomuscular system [17], as can be seen in Figure 1A [18]. The simplified control loop related to the cerebral cortex and cerebellum in supervising the spinal cord’s control of the skeletomuscular system is depicted in Figure 1B [16]. Motor programs and commands are generated in the cerebral cortex, and the motor program is fed into the cerebellum, which sends out motor commands combining programs from the cerebral cortex and the sensory information from the spinal cord. The motor commands from the cerebral cortex and the cerebellum are then summed and sent out to the muscle via the brain stem and the spinal cord. The structure of the human motor control loop gives us some insights into how the CNS controls body movements.

Mimicking the CNS, the CBMC we proposed is shown in Figure 1C. It comprises four parts: the cerebral motor cortex module (CMCM), the cerebral sensory cortex module, the cerebellum module, and the spinal cord module. The spinal cord module carries signals between the arm and the brain and, at the same time, controls some reflexes without involving the brain. Sensory feedback signals from the spinal cord module are then processed in the cerebral sensory cortex module and fed into the CMCM and the cerebellum. The CMCM can choose the appropriate actions and plan the trajectory’s shape to finish the general target. In contrast, the cerebellum module, which is supervised by the CMCM, provides corrections to compensate for errors from nonlinearities, delays, Coriolis, etc., and ensures the smoothness of movement. Motion trajectory is generated in the CMCM by a planner and fed into the agent, modeled by an ANN, and into the cerebellum module, which is in the structure of a cerebellar-like SNN. By taking the trajectory from the planner and the sensory feedback information from the cerebral sensory cortex module, the agent will provide a cerebral torque and instructive inputs to the cerebellum module. In the cerebellum module, the sensory feedback, planned trajectory, and instructive inputs are combined and analyzed for a cerebellum torque response, which is then added with the cerebral torque to form a joint torque command that will be sent down to the spinal cord module. Finally, after being processed in the spinal cord module, the joint torque command is conveyed toward the robot for manipulation.

### 2.1. Cerebellum Module

To better demonstrate how the cerebellum module works, we will introduce it from three perspectives: the neuron model, the synaptic plasticity model, and the network structure.

#### 2.1.1. Neuron Model

In an SNN, neural information transmitted between different neurons is carried in spike sequences, which can be defined as

(1) $$S\left(t\right)=\sum_{f}\delta \left(t_{f}-t_{0}\right),$$

where f=1,2,⋯ is the index label of a spike and δ(·) is a Dirac function. The input signal i(t) of a neuron from one synapse induced by a spike sequence can therefore be described as [19]

(2) $$i\left(t\right)=\int_{0}^{\infty }S\left(s-t\right)exp\left(-s/\tau_{s}\right)ds,$$

where $\tau_{s}$ is a time constant.

In the existing literature, many spiking neural models have been proposed, such as the Hodgkin–Huxley [20] model as well as the Integrate-and-Fire model and its variants [21]. Although the Hodgkin–Huxley model has a better biomimetic reality, it is difficult to realize in the application for computation complexity. Maintaining the feature of membrane potential leakage in neurons and having a high computation efficiency, the Leaky-Integrate-and-Fire (LIF) model [22] is used as the spiking neuron model. The membrane potential of a LIF neuron *u* changes according to

(3) $$\tau_{m}\frac{du}{dt}\left(t\right)=u_{reset}-u\left(t\right)+R\left(i_{0}\left(t\right)+\sum w_{j}i_{j}\left(t\right)\right)$$

where $\tau_{m}=RC$ is the time constant of the neuron membrane that models the voltage leakage. $u_{reset}$ is the potential value after each reset. $i_{0}\left(t\right)$ stands for an external current driving the neural state, $i_{j}\left(t\right)$ denotes the input current from the *j*-th synapse, and $w_{j}$ represents the strength of the *j*-th synapse. Once the membrane potential reaches the firing threshold $u_{fire}$, a single spike is fired from the neuron, and its potential is set back to $u_{reset}$.

#### 2.1.2. Synaptic Plasticity Model

As seen in Equation (3), the neuron potential is influenced by the input synaptic weight $w_{j}$, which can be changed during the working process of the network. How to map the relationship between neuronal activity and the synaptic weights is what synaptic plasticity models will solve. Popular models can be classified into two types, namely, the rate-based and the spike-based. The latter has shown promising applications in robots and other autonomous systems [23], so we will take it here as an example.

The spike-based learning rule, often termed STDP, connects the weight change with the timing of individual spikes. If a presynaptic spike precedes a postsynaptic spike, then the synaptic activity will be strengthened, but if they happen in reversed order, then the synaptic activity will be weakened. The mathematical model of STDP can be given as [24]

(4) $$\Delta w=\left\{\begin{array}{cc} Ae^{\frac{-(|t_{pre}-t_{post}|)}{\tau_{+}}}, & t_{pre}-t_{post}\leq 0\\ Be^{\frac{-(|t_{pre}-t_{post}|)}{\tau_{-}}}, & t_{pre}-t_{post}>0 \end{array}\right.$$

where $t_{pre}$ and $t_{post}$ are the firing time of presynaptic neuron and postsynaptic neuron respectively, $\tau_{+}$ ans $\tau_{-}$ are time constants, and A>0 and B<0 are constants scaling the change of weights, respectively.

#### 2.1.3. Network Structure

**Figure 2:**
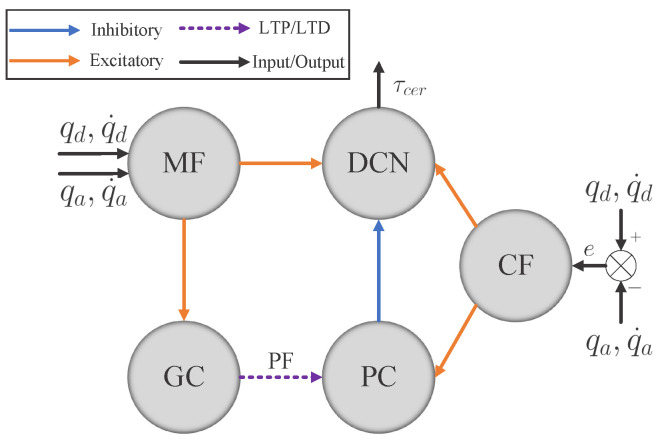
The cerebellar-like network.

Many computational models of the cerebellum have already been proposed, such as CMAC [25] and the Schweighofer–Arbib model [26]. The cerebellar-like network employed in our work is similar to that in [14,15], as depicted in Figure 2.

There are five different neural layers in this network: (1) mossy fibers (MFs); (2) granule cells (GCs); (3) climbing fibers (CFs); (4) Purkinje cells (PCs); (5) deep cerebellar nuclei (DCN). The desired and actual joint position and velocity are concatenated and coded into spiking patterns in the MF layer, which will then project excitatory afferent on both GC and DCN. The movement error of each joint will also be fed into CF and coded into spikes. GC will store and process the spiking pattern from MF and then generate spikes through parallel fibers (PFs) to PC. By combining the neural spike activity of both CF and GC, PC will accordingly give inhibitory afferent to DCN. Finally, joint torque commands will be produced by DCN combining spike information from MF, CF, and PC. The learning ability of this cerebellar-like network is achieved in the PFs by STDP.

### 2.2. Cerebral Motor Cortex Module

The CMCM is constructed of a feed-forward neural network, whose two main purposes are mimicking the dopamine mechanisms in baby learning and supervising the cerebellum module.

#### 2.2.1. Learning Mechanism

The cerebral motor cortex plays an important role in human motion learning through trial and error, especially in babyhood. One of the complex learning mechanisms is induced by dopamine, which facilitates humans to replay newly acquired motions. The principle that humans learn from the consequences of their actions nowadays has been developed as the reinforcement learning (RL) method in artificial intelligence [27]. Therefore, RL is used to mimic the learning mechanism in CMCM, and the whole process can be modeled as a Markov decision process [28]:

(5) $$P\left(s_{t+1}|s_{t}\right)=P\left(s_{t+1}|s_{1},\cdots ,s_{t}\right).$$

The agent (cerebral motor cortex module) selects an action $a_{t}$ at each time step with state $s_{t}$ and policy π

(6) $$a_{t}=\pi \left(s_{t}\right).$$

Then, the next state $s_{t+1}$ is governed by a deterministic transition process

(7) $$s_{t+1}=f\left(s_{t},a_{t}\right)$$

and a reward $r_{t+1}$ is returned from the state $s_{t+1}$ with reward function

(8) $$r_{t+1}=R\left(s_{t+1}\right).$$

Basically, the target of RL is to learn an optimal policy $\pi^{★}$ at each time step to obtain a maximum cumulative reward

(9) $$R_{t}=\sum_{k=1}^{\infty }\gamma^{k-1}r_{t+k},$$

where γ∈[0,1) because earlier rewards are more predictable than the long-term future reward, and the discount rate value helps avoid infinite returns in loopy Markov processes [29].

#### 2.2.2. Supervision to the Cerebellum

As depicted in Figure 1C, the cerebellum module receives an instructive input from the CMCM, which influences the spiking firing rates of neurons in the cerebellum module and serves as supervision of the cerebellum to achieve a specific target. For instance, the CMCM will learn an additional movement to counteract the external force disturbances. A similar scene has been found in the cortex activities of monkeys when learning an arm-reaching task in a curl force field [30]. In addition, to demonstrate the different control levels between the CMCM and cerebellum module, a lower update frequency is employed in the CMCM.

## 3. Case Study: Trajectory Tracking Control of a 7-DoF Robotic Arm

### 3.1. Control Framework

**Figure 3:**
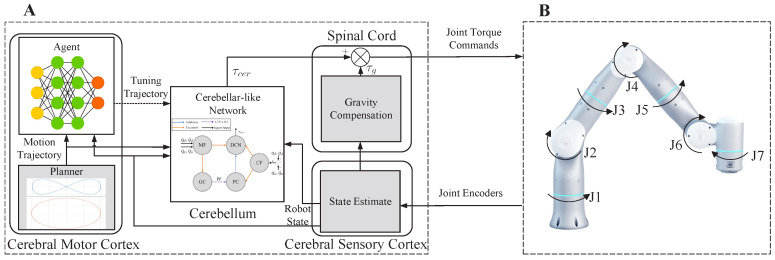
CBMC for the trajectory tracking of a 7-DoF robotic arm. (**A**) Overall control framework of CBMC. (**B**) The diagram of the 7-DoF robotic arm model.

In this section, we apply our method to trajectory tracking control tasks of a 7-DoF robotic arm, the Flexiv Rizon4s, as shown in Figure 3B. The whole control scheme is depicted in Figure 3A, where the direct output from the CMCM to the spinal cord module is omitted, and the dotted line implies a different frequency from other modules. In each control loop of the cerebellum module, the manipulator planner generates predefined reference position and orientation trajectories, and the joint trajectories are calculated with inverse kinematics. The cerebellum module receives the desired joint trajectories $q_{d},{\dot{q}}_{d}$ and feedback states $q_{a},{\dot{q}}_{a}$, then generates joint torques $\tau_{cer}$. The spinal cord module here provides a gravity compensation torque. With every 20 loops of the cerebellum module running, the CMCM updates an additional zero-order-hold instructive input added to the desired joint trajectories based on the current targets and robot states.

### 3.2. Implementation of CBMC

#### 3.2.1. Cerebellum-like SNN

In this paper, the cerebellum module is implemented with SpikingJelly [31], which is an open-source deep learning framework for SNN and has been used for exploring the applications of bio-inspired SNN in many aspects [32,33,34]. The cerebellum-like SNN described in Figure 2 consists of five layers: MFs, GCs, PCs, CFs, and DCN. All of them are divided into seven microcomplexes, each one for controlling a robot joint. In the MF–GC, CF–PC, CF–DCN, and PC–DCN connections, the seven microcomplexes are indeed independent, where the MF–GC, CF–PC, and CF–DCN connections act like encoders. However, the neurons from MFs to DCN and GCs to PCs are all fully connected, which means the seven microcomplexes are dependent. The MF–DCN connection generates a constant membrane voltage changing to both positive and negative torque neurons in DCN, which helps in reducing the noise influence. The GC–PC connection is where STDP learns the dynamic mechanism of the robot from the command information encoded in MF and the error information encoded in CF and improves the control effects of the cerebellum-like SNN. The following part will introduce how to implement the five layers in detail.

The MF layer has 40 spiking neurons per microcomplex, 280 in total, translating the analog information to spikes. For each joint, the 40 neurons are divided into four subgroups for encoding feedback and desired joint positions and velocities, respectively, with ten neurons each. For an analog value *a* with interval $\left[r_{\min},r_{\max}\right]$, one spike $S_{i,\mathrm{MF}}\left(i=1,2,\cdots ,10\right)$ among the 10 neurons will be fired when

(10) $$\begin{array}{c} a\in [c_{i-1},c_{i}],\\ c_{k}=r_{\min}+k\;\cdot \;\frac{\left(r_{\max}-r_{\min}\right)}{10},k=0,1,\cdots ,10. \end{array}$$

Therefore, 4 neurons per joint and 28 in total will be active at each time step. Every combination of four spikes is uniquely connected to one of 10,000 neurons per microcomplex in the GC layer with the excitatory synapse, represented by a positive weight $w_{\mathrm{MF}-\mathrm{GC}}$. All the neurons in the MF layer are concatenated together, fully connecting to the neurons in the DCN layer with excitatory synapse weight $w_{\mathrm{MF}-\mathrm{DCN}}$.

CF layer modifies the error between the desired and actual trajectories per joint to spikes with 100 spiking neurons per microcomplex. The front half of the 100 neurons are dedicated to the forward movement of each joint, and the back half are for joint reversing, which mimics the interaction between agonist and antagonist muscles in human movement. The normalized error value $e_{j}\in \left[-1,1\right]$ of each joint is given as

(11) $$e_{j}=\frac{q_{d,j}-q_{a,j}+{\dot{q}}_{d,j}-{\dot{q}}_{a,j}}{q_{\mathrm{upper},j}-q_{\mathrm{lower},j}+{\dot{q}}_{\mathrm{upper},j}-{\dot{q}}_{\mathrm{lower},j}},\;j=1,2,\cdots ,7,$$

where $q_{d,j},{\dot{q}}_{d,j},q_{a,j},{\dot{q}}_{a,j}$ are the desired and actual joint position and velocity, respectively, and $q_{\mathrm{upper},j},q_{\mathrm{lower},j},{\dot{q}}_{\mathrm{upper},j},{\dot{q}}_{\mathrm{lower},j}$ are the upper and lower bounds of *j*-th joint position and velocity. Poisson encoding is applied depending on the error value of each joint to obtain the spikes $S_{j,i,\mathrm{CF}}$, which can be expressed as

(12) $$S_{j,i,\mathrm{CF}}=\left\{\begin{array}{cc} 1, & \mathrm{if}\;|e_{j}|>\mathrm{rand}\left(0,1\right)\\ 0, & \mathrm{else}. \end{array}\right.,\;j=1,2,\cdots ,7.$$

In order to be consistent with the joint movement, only up to half of the neurons of the CF layer will be active per microcomplex, which means if $e_{j}>0$, i=1,2,⋯,50; otherwise, i=51,52,⋯,100. Each neuron in the CF layer is connected one-to-one with each neuron in the PC layer and DCN layer with excitatory synapse weights $w_{\mathrm{CF}-\mathrm{PC}}$ and $w_{\mathrm{CF}-\mathrm{DCN}}$, respectively, also indicating the two other layers have the same number of neurons with the CF layer.

Neurons in the GC, PC, and DCN layers are all modeled as discrete-time LIF neurons to approximate the dynamics of the continuous-time LIF neurons. The membrane potential discrete-time charging function of the LIF neuron is

(13) $$h\left[t\right]=v\left[t-1\right]-\frac{1}{\tau_{m}}\left(v\left[t-1\right]-v_{reset}\right)+x\left[t\right],$$

where $\tau_{m}$ is the voltage leaking time constant and x[t] is the input from synapses. To avoid confusion, h[t] is used to represent the membrane potential after neuronal charging but before neuronal firing at time *t*, v[t] is the membrane potential after neuronal firing, and $v_{reset}$ is the reset value of membrane potential. The reset function of the membrane potential v[t] depending on the firing state is

(14) $$v\left[t\right]=\left\{\begin{array}{cc} v_{reset}, & \mathrm{if}\;S[t]=1\\ h[t], & \mathrm{else}. \end{array}\right.$$

The firing state of the LIF neuron is described as

(15) $$S\left[t\right]=\left\{\begin{array}{cc} 1, & \mathrm{if}\;h\left[t\right]\geq v_{fire}\\ 0, & \mathrm{else}. \end{array}\right.,$$

where $v_{fire}$ is the firing threshold. Therefore, a LIF neuron will fire a spike when the membrane potential h[t] reaches the firing threshold. All the configuration parameters of LIF neurons are summarized in Table 1.

The control mechanism of the cerebellum module is learned at the GC–PC connections by adjusting the synapses in PFs with the STDP mechanism. The trace method [35] is used to implement STDP and avoid recording all the firing times of presynaptic and postsynaptic neurons described in Equation (4). The update of synapse weight at time *t* with the trace method is

(16) $$\Delta w_{i,j}\left[t\right]=f_{post}\left(w_{i,j}\left[t\right]\right)\;\cdot \;tr_{i}\left[t\right]\;\cdot \;S_{j}\left[t\right]-f_{pre}\left(w_{i,j}\left[t\right]\right)\;\cdot \;tr_{j}\left[t\right]\;\cdot \;S_{i}\left[t\right],$$

where indices i,j indicate the presynaptic and postsynaptic neurons, respectively, $f_{post},f_{pre}$ are functions constraining how weight changes, and $tr_{i}\left[t\right],tr_{j}\left[t\right]$ are the traces of the presynaptic and postsynaptic neurons that track their firing. The updated functions of the traces are

(17) $$\begin{array}{cc} tr_{i}\left[t\right] & =tr_{i}\left[t-1\right]-\frac{tr_{i}\left[t-1\right]}{\tau_{pre}}+S_{i}\left[t\right]\\ tr_{j}\left[t\right] & =tr_{j}\left[t-1\right]-\frac{tr_{j}\left[t-1\right]}{\tau_{post}}+S_{j}\left[t\right], \end{array}$$

where $\tau_{pre},\tau_{post}$ are the time constants of the presynaptic and postsynaptic neurons, similar to the leakage of LIF neurons. $S_{i}\left[t\right],S_{j}\left[t\right]$ in both Equations (16) and (17) are the firing states of the presynaptic and postsynaptic neurons.

Receiving excitatory synapses from the GC and CF layers, the neurons in the PC layer are activated and then one-to-one connected to the neurons in the DCN layer but with an inhibitory synapse, represented by a negative weight $w_{\mathrm{PC}-\mathrm{DCN}}$. Table 2 summarizes all the synapse weights. Finally, combining all the excitatory synapses from MF and CF layers and inhibitory synapses from the CF layer, the neurons in the DCN layer generate spikes, and then those spikes are mapped to joint torques $\tau_{cer}$. The decode function of each microcomplex is as follows

(18) $$\tau_{cer,j}=\alpha_{j}\left(\sum_{i=1}^{50}S_{j,i}\left[t\right]-\sum_{i=51}^{100}S_{j,i}\left[t\right]\right),$$

where j=1,2,⋯,7 is corresponding to the joint number, $\alpha_{j}$ is the mapping factor transforming the spikes to torques and is set as α=(4.5,4.5,4.5,1.7,2.7,1.0,0.05)N·m/spike.

#### 3.2.2. CMCM with Deep Deterministic Policy Gradient

In this project, deep deterministic policy gradient (DDPG) [36] algorithm as the RL implementation in CMCM is adopted to supervise the cerebellum module, based on a deep reinforcement learning library PFRL [37]. DDPG is a model-free algorithm that learns the deterministic policy to the continuous action domain, as

(19) $$a=\mu (s|\theta^{\mu })$$

where $a\in R^{7}$ is the action vector, $s\in R^{28}$ is the state vector, and $\theta^{\mu }$ is the parameter of the policy network. The actions are interpreted as additional desired joint positions and added to the original position targets from the trajectory generator. The state vector s is spliced by the desired and actual joint positions and velocities.

In addition, an action-value function Q(s,a) is used in DDPG for describing the expected reward in Equation (9) after taking an action $a_{t}$ in state $s_{t}$. Considering the function approximators parameterized by $\theta^{Q}$, one target of the DDPG is minimizing the Bellman residual

(20) $$L\left(\theta^{Q}\right)=E\left[{\left(Q\left(s_{t},a_{t}|\theta^{Q}\right)-y_{t}\right)}^{2}\right],$$

where

(21) $$y_{t}=r\left(s_{t},a_{t}\right)+\gamma Q^{\prime }\left(s_{t+1},\mu^{\prime }\left(s_{t+1}|\theta^{\mu^{\prime }}\right)|\theta^{Q^{\prime }}\right).$$

Here, $r(s_{t},a_{t})$ is the reward function and γ is the discount factor, $Q^{\prime },\mu^{\prime }$ are target networks. Another target of the DDPG is learning the policy, which is evaluated by maximizing the performance objective

(22) $$J\left(\theta^{\mu }\right)=E\left[Q(s_{t},\mu \left(s_{t}|\theta^{\mu }\right))\right].$$

For the trajectory-tracking tasks, the total reward *f* in one simulation step is a weighted sum of the punishment of the joint errors and Cartesian position error as

(23) $$f=0.5\sum_{j=1}^{7}f_{joint,j}+f_{c},$$

where

(24) $$\begin{array}{cc} f_{joint,j} & =\left\{\begin{array}{cc} -0.1 & ,\mathrm{if}\;{\dot{q}}_{des,j}\;\cdot \;{\dot{q}}_{act,j}<0\\ -10∥q_{des,j}-q_{act,j}{∥}^{2} & ,\mathrm{else}. \end{array}\right.\\ f_{c} & =-10∥x_{des}-x_{act}{∥}^{2}. \end{array}$$

A constant punishment is given if the desired and actual joint velocity direction are not the same. Otherwise, we punish the joint position errors. Here, x denotes the Cartesian position of the end-effector, and the punishment is set as the distance from the target to the estimated Cartesian position.

The whole training process is divided into two stages. First, the cerebellum module is pre-trained without the CMCM, then it is fixed, and the agent explores the tuning policy to the cerebellum module with the aid of the reward mechanism. The learning algorithm of CMCM is as shown in Algorithm 1.

**Figure 4:**
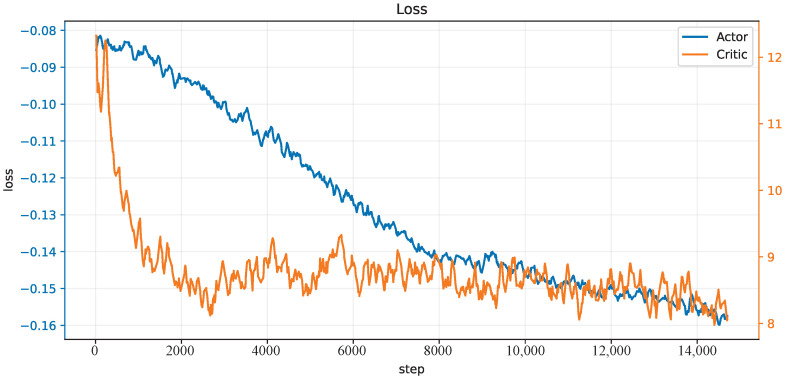
The loss curves of the actor and critic networks.

We train our CBMC with a specific trajectory target, which is an inclined circle as described in Equation (25), and without payload on the end-effector in the PyBullet physics simulator [38], and 150 trials in each epoch. The initial state of the robot is not on the trajectory at the beginning. One hundred epochs, thus 15 k trials, are performed, and the learning curves of the actor network and critic network are shown in Figure 4. After this learning process, the controller is applied to different trajectory-tracking tasks and is faced with unknown payloads on the end-effector.
**Algorithm 1** Learning algorithm of CBMC1:Load the cerebellum-like SNN2:Initialize main critic network, actor network, target networks, and replay buffer3:Initialize relative frequency *F* between cerebellum module and CMCM4:**for** epoch =1 to *N* **do**5:   Initialize the neurons states6:   Initialize the robot states7:   Initialize the period reward r=08:   **for** t=1 to *M* **do**9:     **if** (*t* Mod *F*) == 1 **then**10:        Generate action $a_{t}$ according to current policy and states11:        Update cerebellum-like SNN12:    **end if**13:    Calculate torque commands τ from CBMC14:    Execute torque commands and observe new states $s_{t+1}$15:    Compute reward *f* and accumulate the period reward r=r+f16:    **if** (*t* Mod *F*) == 1 and t≠1 **then**17:        Store transition $\left(s_{t-F},a_{t-F},R,s_{t}\right)$ in replay buffer18:        Reset the period reward r=019:        Update the critic and actor networks by training on a small batch of samples from the replay buffer20:        Update the target networks21:    **end if**22:   **end for**23:**end for**

### 3.3. Experiment Settings

To assess the efficacy of our novel control strategy in robot dynamic control and trajectory tracking, we execute experiments considering two key factors. On the one hand, we test our CBMC on the robotic arm with different payloads on the end-effector in smooth trajectories. A single-joint movement will cause interaction forces to all other joints. The disturbance force cannot be compensated easily on the condition that the dynamics model is unknown. On the other hand, we test our CBMC controlling the end-effector tracking different trajectories containing a circle trajectory in the inclined plane and an eight-like trajectory in the horizontal plane, covering most of the possible translation motions of the robotic arm in the Cartesian space. The circular and eight-like trajectories are described in Equation (25).

(25) $$\mathrm{Circle}:\left\{\begin{array}{cc} x & =x_{0}+R_{c}\;\cdot \;\cos\left(2\pi t/T_{c}\right)\;\cdot \;\cos\theta \\ y & =y_{0}+R_{c}\;\cdot \;\sin\left(2\pi t/T_{c}\right)\\ z & =z_{0}+R_{c}\;\cdot \;\cos\left(2\pi t/T_{c}\right)\;\cdot \;\sin\theta \end{array}\right.\;\mathrm{Eight}-\mathrm{like}:\left\{\begin{array}{cc} x & =x_{0}+R_{e}\;\cdot \;\cos\left(2\pi t/T_{e}\right)\\ y & =y_{0}+0.5R_{e}\;\cdot \;\sin\left(4\pi t/T_{e}\right)\\ z & =z_{0} \end{array}\right.,$$

where $R_{c}=R_{e}=0.14\;m$ is the radius of the trajectory, $T_{c}=T_{e}=3s$ is the period, and $\theta =30{}^{\circ }$ is the slant angle of the circle along the horizontal plane. $\left(x_{0},y_{0},z_{0}\right)$ is used to adapt the trajectory within the workspace of robot. Providing the 3-D position and maintaining the orientation of the end-effector, the joint trajectories are calculated through an offline process using the inverse kinematics of the robot.

The performance of the CBMC on trajectory tracking is evaluated by comparing the desired and the actual joint positions. We use the mean square error (MSE) as the metric to evaluate the errors described in the following equations:

(26) $$\begin{array}{cc} {\mathrm{MSE}}_{j} & =\frac{1}{K}\sum_{t=1}^{K}{∥q_{d,j}\left[t\right]-q_{a,j}\left[t\right]∥}^{2}\\ \mathrm{MSE} & =\frac{1}{N}\sum_{j=1}^{N}{\mathrm{MSE}}_{j}, \end{array}$$

where $K=3\times 10^{4}$ denotes the simulation timestep number, corresponding to 10 cycles of the trajectories, and N=7 is the number of joints.

## 4. Results and Discussion

In this section, we outline the experimental results that show how our method works and verify its effectiveness in trajectory tracking facing unknown payloads. To demonstrate that, the performances of the control effects with payloads of 0, 0.5, and 2.5 kg are studied on the aforementioned inclined circle and eight-like trajectories. On the other hand, we also evaluate our controller in target reaching task, whose movement is an s-curve toward a target point over time, to show the ability to face irregular but usual movements in human daily life.

**Figure 5:**
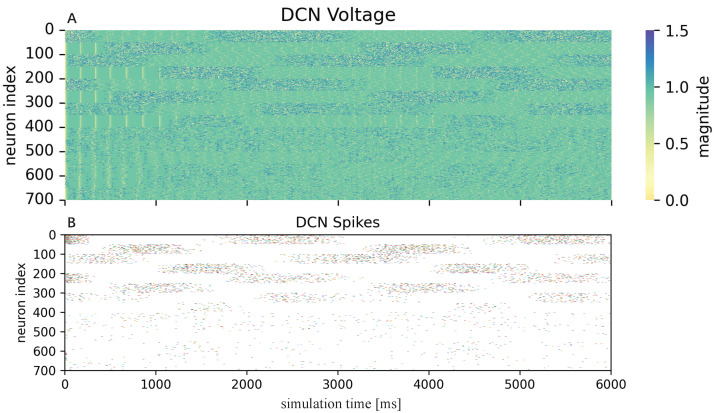
The DCN neurons’ activities in the first three cycles under the condition of inclined circle trajectory and no payload. (**A**) The heat map of the membrane voltages of the DCN neurons. (**B**) The corresponding spike’s firing states.

Firstly, a brief description of the neuron activities in the control process is described in Figure 5, which shows the DCN neurons’ activities in the first three cycles under the condition of inclined circle trajectory and no payload on the end-effector. There are seven hundred neurons, and each hundred corresponds to a joint actuator. When the membrane voltage of a neuron reaches its firing threshold, which is set as 1.5 in Figure 5A, one can see a corresponding spike is fired in Figure 5B.

**Figure 6:**
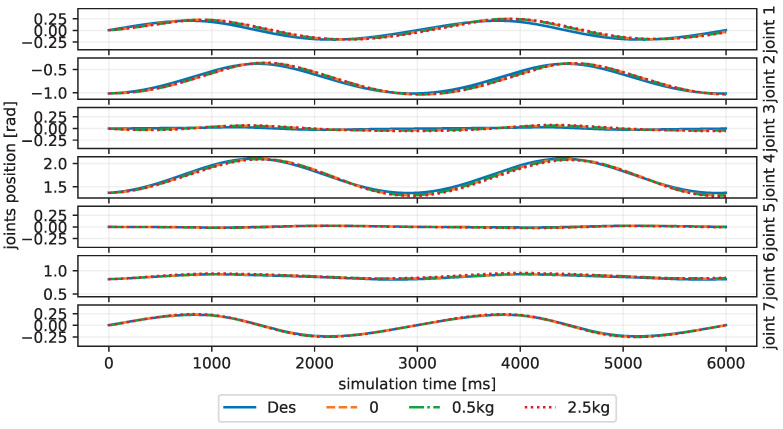
The joint trajectories tracking curves of the CBMC to different payloads in the first three cycles under the inclined circle trajectory condition.

Taking the first joint as an example, the first fifty neuron spikes will generate a positive acceleration and, therefore, dense spikes are fired in the beginning time as shown in Figure 5B to accelerate the robotic arm from a static state to the desired trajectory. In contrast, the last fifty neuron spikes will generate a deceleration by negative torque. Thus, combined with the orange dotted line in Figure 6, which is the corresponding joint tracking trajectory, indicating deceleration of the first joint around 800, 3800 ms and acceleration around 2200, 5200 ms, we find it is consistent with the DCN neuron spikes’ activity as shown in Figure 5B, where dense spikes of the last fifty neurons are fired when there is deceleration and the first fifty are fired when there is acceleration. This phenomenon is not obvious in the last three joints because of the small torques for the same joint movements.

**Figure 7:**
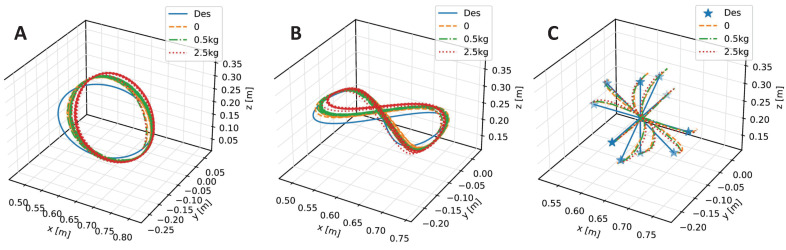
The desired versus actual trajectories of the end-effector with different payloads in the Cartesian space. (**A**) The inclined circle trajectory in Cartesian space. (**B**) The 8-like trajectory in Cartesian space. (**C**) The target reaching task trajectory in Cartesian space.

Different payloads, as mentioned before, are tested on the inclined circle trajectory. Figure 6 shows the joint trajectories tracking curves of the CBMC with different payloads in the first three cycles, and Figure 7A shows end-effector tracking curves in the whole ten cycles. The results show the reliable ability of the CBMC when the dynamics of the robotic arm change.

**Figure 8:**
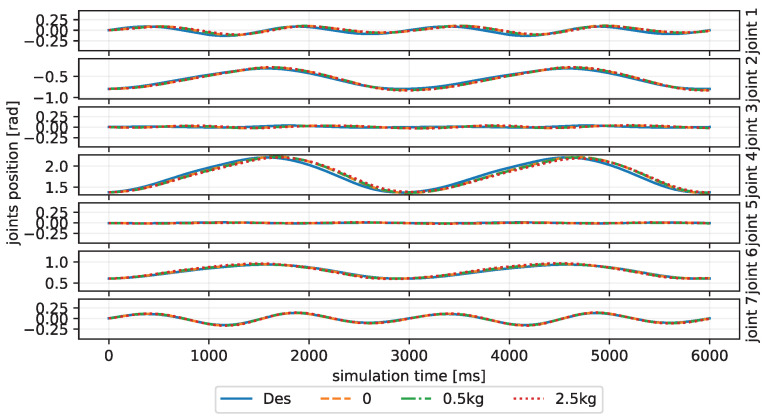
The joint trajectories tracking curves of the CBMC to different payloads in the 8-like trajectory condition.

In addition, we also test the CBMC on the eight-like trajectory that it never learns in the training process. Compared to the circle trajectory, the eight-like trajectory requires a faster and steeper change of the velocity and direction in the Cartesian space, resulting in more joint disturbances. Nonetheless, as depicted in the Cartesian trajectories of Figure 7B and the joint trajectories of Figure 8, the CBMC still shows a good performance on the condition of unknown trajectory and payloads. Table 3 lists the MSE of the joint position error under different trajectories and payloads. The CBMC shows a similar MSE loss in the Eight-like trajectory of about $1.2\times 10^{-4}$ compared to the training inclined circle trajectory for all conditions of payloads.

The target-reaching task consists of ten different reaching targets as the star markers shown in Figure 7C, which are around the same starting point. The challenges are interaction forces caused by acceleration and deceleration at the beginning and end, and the irregular directions toward different targets. Nevertheless, the CBMC performs a capable result in the target-reaching task from the Cartesian trajectory as shown in Figure 7C and the MSE in Table 3.

Finally, to demonstrate the effectiveness of the CBMC in dealing with unknown dynamics changes, we compare it with a PD controller on joint space. The PD controller is designed to have a similar (and even a little better) performance with CBMC on the inclined circle trajectory-tracking task and with no payload, the condition in which the CBMC is trained. Table 4 lists the MSE of different methods on inclined circle and with different payloads, where the method “No CMCM” corresponds to our method but removing the instructive input from CMCM. When the payload is added to the end-effector, we can see the PD controller performs worse than the CBMC. Particularly for the payload of 2.5 kg, the MSE of PD increases about 52% compared to the CBMC. Combining the MSE of the “No CMCM”, we can conclude that both the instructive inputs from CMCM and the dynamics mechanism learned in cerebellum module cause the CBMC have a better performance on this problem. In addition, the comparison between the CBMC and “No CMCM” implies the contribution of the supervising mechanism from the CMCM to the cerebellum module.

## 5. Conclusions and Future Work

In this paper, inspired by the human control loop that outlines the CNS, we propose the CBMC approach, which mainly consists of four parts: the cerebral motor cortex module, the cerebellum module, the cerebral sensory cortex module, and the spinal cord module. Mimicking the biological feature in the human motor control system, the cerebellum module constructed by SNN aims to learn the dynamics feature of the robot, and it is supervised by instructive inputs from the cerebral motor cortex module, which learns using RL. The cerebral sensory cortex module deals with feedback information, including self-perception and environment interaction, while the spinal cord module modulates torque commands.

The proposed method was applied to controlling a 7-DOF robotic arm and partially simplified in the trajectory-tracking task, where the DDPG was used as the RL algorithm in the cerebral motor cortex module and the cerebellum-like SNN was implemented in the cerebellum module. To validate its effectiveness, we firstly trained the CBMC in a specific inclined circle trajectory-tracking task with no payload on the end-effector, then we verified its performances on the condition of different payloads and a new eight-like trajectory. Finally, we compared it to a PD controller to demonstrate the effectiveness of the supervising mechanism and the cerebellum-like SNN.

One limitation of this work is that the method is only validated in the simulation because the spiking neuron model with Python is not feasible for temporary torque control in the real robot manipulator. In the future, we will develop the proposed approach on real robotic arms. In addition, the ability of the CMCM can be explored in more complex tasks combining the cerebral sensory cortex module, like interacting with the environment, and the spinal cord module can be considered to control the rhythmic motion as a part of the whole motion control system.

## Figures and Tables

**Table 1:** Model parameters of discrete-time LIF neurons.

Parameters	GC	PC	DCN
$v_{reset}$	0	0	0
$v_{fire}$	1.0	5.0	1.5
$\tau_{m}$	50	60	12

**Table 2:** Parameters of synapse weights.

Synapses	$w_{\mathrm{MF}-\mathrm{GC}}$	$w_{\mathrm{MF}-\mathrm{DCN}}$	$w_{\mathrm{CF}-\mathrm{DCN}}$	$w_{\mathrm{CF}-\mathrm{PC}}$
value	0.25	0.0028	0.45	−0.5

**Table 3:** MSE of the joint position error under different trajectories and payloads.

Trajectory	No Payload $\left(\times 10^{-4}\right)$	0.5 kg $\left(\times 10^{-4}\right)$	2.5 kg $\left(\times 10^{-4}\right)$
Inclined Circle	7.134±0.749	8.250±1.075	12.825±1.795
Eight-Like Trajectory	8.340±0.725	9.642±0.819	13.862±0.934
Target Reaching	1.266±0.646	1.309±0.711	1.633±0.936

**Table 4:** MSE of different payloads in inclined circle: comparing to different methods.

Methods	No Payload $\left(\times 10^{-4}\right)$	0.5 kg $\left(\times 10^{-4}\right)$	2.5 kg $\left(\times 10^{-4}\right)$
No CMCM	7.845±0.898	8.883±1.021	14.064±1.805
CBMC	7.134±0.749	8.250±1.075	12.825±1.795
PD	7.036±0.219 ^1^	8.740±0.356	19.496±1.989

^1^ The bold number implies the minimum MSE under the corresponding payload condition.

## Data Availability

No new data were created or analyzed in this study. Data sharing is not applicable to this article.

## Abbreviations

The following abbreviations are used in this manuscript:

DoF	Degree of Freedom
ANN	artificial neuron network
CNS	central nervous system
CBMC	CNS-based Biomimetic Motor Control
SNN	spiking neural network
RL	reinforcement learning
STDP	spiking timing-dependent plasticity
CMCM	cerebral motor cortex module
LIF	Leaky-Integrate-and-Fire
MF	mossy fiber
GC	granule cell
CF	climbing fiber
PC	Purkinje cell
DCN	deep cerebellar nuclei
PF	parallel fiber

## References

[1] Chaoui H., Sicard P., Gueaieb W. (2009). ANN-Based Adaptive Control of Robotic Manipulators with Friction and Joint Elasticity. IEEE Trans. Ind. Electron..

[2] He W., Dong Y. (2018). Adaptive Fuzzy Neural Network Control for a Constrained Robot Using Impedance Learning. IEEE Trans. Neural Netw. Learn. Syst..

[3] Wang F., Liu Z., Chen C.L.P., Zhang Y. (2018). Adaptive neural network-based visual servoing control for manipulator with unknown output nonlinearities. Inf. Sci..

[4] Salloom T., Yu X., He W., Kaynak O. (2020). Adaptive Neural Network Control of Underwater Robotic Manipulators Tuned by a Genetic Algorithm. J. Intell. Robot. Syst..

[5] Liu C., Zhao Z., Wen G. (2019). Adaptive neural network control with optimal number of hidden nodes for trajectory tracking of robot manipulators. Neurocomputing.

[6] Pham D.T., Nguyen T.V., Le H.X., Nguyen L.V., Thai N.H., Phan T.A., Pham H.T., Duong A.H. (2019). Adaptive neural network based dynamic surface control for uncertain dual arm robots. Int. J. Dyn. Control.

[7] Liu Z., Peng K., Han L., Guan S. (2023). Modeling and Control of Robotic Manipulators Based on Artificial Neural Networks: A Review. Iran. J. Sci. Technol. Trans. Mech. Eng..

[8] Chadderdon G.L., Neymotin S.A., Kerr C.C., Lytton W.W. (2013). Correction: Reinforcement Learning of Targeted Movement in a Spiking Neuronal Model of Motor Cortex. PLoS ONE.

[9] Spüler M., Nagel S., Rosenstiel W. A spiking neuronal model learning a motor control task by reinforcement learning and structural synaptic plasticity. Proceedings of the 2015 International Joint Conference on Neural Networks (IJCNN).

[10] Bouganis A., Shanahan M. Training a spiking neural network to control a 4-dof robotic arm based on spike timing-dependent plasticity. Proceedings of the The 2010 International Joint Conference on Neural Networks (IJCNN).

[11] Chen X., Zhu W., Dai Y., Ren Q. A bio-inspired spiking neural network for control of a 4-dof robotic arm. Proceedings of the 2020 15th IEEE Conference on Industrial Electronics and Applications (ICIEA).

[12] DeWolf T., Patel K., Jaworski P., Leontie R., Hays J., Eliasmith C. (2023). Neuromorphic control of a simulated 7-DOF arm using Loihi. Neuromorphic Comput. Eng..

[13] Carrillo R.R., Ros E., Boucheny C., Olivier J.M.C. (2008). A real-time spiking cerebellum model for learning robot control. Biosystems.

[14] Abadia I., Naveros F., Garrido J.A., Ros E., Luque N.R. (2019). On robot compliance: A cerebellar control approach. IEEE Trans. Cybern..

[15] Abadía I., Naveros F., Ros E., Carrillo R.R., Luque N.R. (2021). A cerebellar-based solution to the nondeterministic time delay problem in robotic control. Sci. Robot..

[16] Van Der Smagt P., Arbib M.A., Metta G. (2016). Neurorobotics: From vision to action. Springer Handbook of Robotics.

[17] Swinnen S.P., Vangheluwe S., Wagemans J., Coxon J.P., Goble D.J., Van Impe A., Sunaert S., Peeters R., Wenderoth N. (2010). Shared neural resources between left and right interlimb coordination skills: The neural substrate of abstract motor representations. Neuroimage.

[18] Bhat A. (2017). A Soft and Bio-Inspired Prosthesis with Tactile Feedback.

[19] Ponulak F., Kasinski A. (2011). Introduction to spiking neural networks: Information processing, learning and applications. Acta Neurobiol. Exp..

[20] Hodgkin A.L., Huxley A.F. (1952). A quantitative description of membrane current and its application to conduction and excitation in nerve. J. Physiol..

[21] Burkitt A.N. (2006). A review of the integrate-and-fire neuron model: I. Homogeneous synaptic input. Biol. Cybern..

[22] Stein R.B. (1965). A theoretical analysis of neuronal variability. Biophys. J..

[23] Bing Z., Meschede C., Röhrbein F., Huang K., Knoll A.C. (2018). A survey of robotics control based on learning-inspired spiking neural networks. Front. Neurorobot..

[24] Gerstner W., Kistler W.M. (2002). Mathematical formulations of Hebbian learning. Biol. Cybern..

[25] Albus J.S. (1975). A new approach to manipulator control: The cerebellar model articulation controller (CMAC). J. Dyn. Sys. Meas. Control.

[26] Schweighofer N. (1995). Computational Models of the Cerebellum in the Adaptive Control of Movements.

[27] Holroyd C.B., Coles M.G.H. (2002). The neural basis of human error processing: Reinforcement learning, dopamine, and the error-related negativity. Psychol. Rev..

[28] Sutton R.S., Barto A.G. (2005). Reinforcement Learning: An Introduction. IEEE Trans. Neural Netw..

[29] Nagaraj A., Sood M., Patil B.M. (2022). A Concise Introduction to Reinforcement Learning in Robotics. arXiv.

[30] Sun X., O’Shea D.J., Golub M.D., Trautmann E.M., Vyas S., Ryu S.I., Shenoy K.V. (2022). Cortical preparatory activity indexes learned motor memories. Nature.

[31] Fang W., Chen Y., Ding J., Chen D., Yu Z., Zhou H., Timothée M., Tian Y. (2020). SpikingJelly. https://github.com/fangwei123456/spikingjelly.

[32] Yuan R., Duan Q., Tiw P.J., Li G., Xiao Z., Jing Z., Yang K., Liu C., Ge C., Huang R. (2022). A calibratable sensory neuron based on epitaxial VO2 for spike-based neuromorphic multisensory system. Nat. Commun..

[33] Xiang S., Zhang T., Jiang S., Han Y., Zhang Y., Du C., Guo X., Yu L., Shi Y., Hao Y. (2022). Spiking SiamFC++: Deep Spiking Neural Network for Object Tracking. arXiv.

[34] Liu G., Deng W., Xie X., Huang L., Tang H. (2022). Human-Level Control through Directly-Trained Deep Spiking Q-Networks. IEEE Trans. Cybern..

[35] Morrison A., Diesmann M., Gerstner W. (2008). Phenomenological models of synaptic plasticity based on spike timing. Biol. Cybern..

[36] Lillicrap T.P., Hunt J.J., Pritzel A., Heess N.M.O., Erez T., Tassa Y., Silver D., Wierstra D. (2015). Continuous control with deep reinforcement learning. arXiv.

[37] Fujita Y., Nagarajan P., Kataoka T., Ishikawa T. (2021). ChainerRL: A Deep Reinforcement Learning Library. J. Mach. Learn. Res..

[38] Coumans E., Bai Y. PyBullet, a Python Module for Physics Simulation for Games, Robotics and Machine Learning. 2016–2021. http://pybullet.org.

